# Patterns of Uveitis in the Middle East and Europe

**Published:** 2011-10

**Authors:** Ebrahim M. Nashtaei, Masoud Soheilian, Carl P. Herbort, Mehdi Yaseri

**Affiliations:** 1 Ophthalmic Research Center, Shahid Beheshti University of Medical Sciences, Tehran, Iran; 2 Inflammatory and Retinal Eye Diseases, Center for Ophthalmic Specialized Care, Lausanne, Switzerland; 3 University of Lausanne, Lausanne, Switzerland

**Keywords:** Uveitis, Europe, Middle East, Patterns of Uveitis

## Abstract

**Purpose:**

To compare the patterns of uveitis, emphasizing similarities and discrepancies, in the Middle East and Europe.

**Methods:**

Six articles reporting uveitis patterns from the Middle East including a total of 2,693 cases, and seven articles with a sum of 4,379 cases from Europe were analyzed and patterns in each region were defined and compared.

**Results:**

In both regions, uveitis was most commonly seen in the fourth decade of life with anterior uveitis being the most common anatomical form. Idiopathic cases accounted for the majority of anterior and intermediate uveitis; toxoplasmosis was the most frequent entity in posterior uveitis while Behcet’s disease and idiopathic forms were the next most common causes in the Middle East and in Europe, respectively.

**Conclusion:**

Since patterns of uveitis differ in various geographic regions, discovering these patterns would be helpful for the diagnosis and treatment of this broad category of conditions. This necessitates applying a universal diagnostic classification system to enable accurate comparisons.

## INTRODUCTION

Uveitis accounts for 5–20% of legal blindness in Europe and the United States^1^, therefore recognition of the prevalence and patterns of this diverse group of ocular disorders is necessary for their diagnosis and treatment.

Due to the varying prevalence of infectious (e.g. tuberculosis and toxoplasmosis) and non-infectious (e.g. Behçet’s disease) entities in different parts of the world, one would expect variability in the prevalence and patterns of uveitis. This variability may be influenced by multiple factors including race and genetic background, environmental factors, referral patterns, and diagnostic criteria and facilities. For instance, in the US, the four most common forms of uveitis include acute anterior uveitis associated with ankylosing spondylitis, sarcoidosis, cytomegalovirus retinitis, and toxoplasmosis.^2^–^5^ In Japan, the incidence of Behçet’s disease, Vogt-Koyanagi-Harada (VKH) syndrome, and toxoplasmosis have shown an apparent decrease, while the incidence of sarcoidosis and acute anterior uveitis are increasing.^6^–^8^

In the Indian subcontinent, toxoplasmosis, VKH syndrome, and idiopathic anterior uveitis are most frequently observed.^9^,^10^ In Africa, AIDS, herpes zoster ophthalmicus, toxoplasmosis, tuberculosis (TB), rheumatoid arthritis, and onchocerciasis are the most commonly associated diseases.^11^

The purpose of this study was to compare patterns of uveitis in Europe with that in the Middle East, and to highlight similarities and disparities.

## METHODS

Six articles^12^–^17^ from the Middle East including a sum of 2,693 cases and seven articles from Europe^18^–^24^ with a total number of 4,379 cases were reviewed and compared. First, articles originating from the Middle East underwent review and data analysis, then they were assessed as a whole and general statistics for this region were extracted. European articles were analyzed similarly followed by comparison of data from the two regions.

Except for the Finnish study^20^, which was community-based, all other reports were obtained from tertiary referral centers. In the study performed in Israel^17^, the criteria for classification of uveitis were not mentioned. Standardization of uveitis nomenclature group (SUN) and the criteria used by Perkins (1961) were applied in Turkey^14^ and Finland^20^ respectively and the rest had used the international uveitis study group (IUSG) criteria for this purpose.

Having been classified as an idiopathic and independent disease in some articles, all cases of pars planitis were considered as idiopathic for uniformity. An article from Israel^17^ only reviewed chronic uveitis cases and therefore did not represent a comprehensive report from that region. Statistical data from some articles were not classifiable at all and were thus excluded from calculations. For example, the Brussels study^22^ did not include anterior uveitis and in studies from Poland^19^, Italy^21^ and Saudi Arabia^15^ the number of cases in anatomic subgroups was not mentioned.

To present data, we used mean ± standard deviations (SDs) and frequencies (percentage). To compute these values we used weighted means proportional to sample size of a particular paper. Whenever there was no information about a particular item, we disregarded that particular data. All statistical analyses were performed using SPSS software version 17.0 (SPSS Co., Chicago, USA).

## RESULTS

Mean age of participants in Middle Eastern and European studies was 35.2 and 39.1 years, respectively. Male subjects comprised 52% of cases in Middle Eastern and 49.4% of subjects in European studies. There was no significant difference in terms of age and sex between Middle Eastern and European studies; both study groups reported the fourth decade of life as the most common age for presentation of uveitis (Table 1).

In studies from the Middle East, the most common anatomical forms, in descending order, were anterior (49.6%), pan- (24.9%), posterior (15.5%), and intermediate uveitis (10.3%); however, in the Ankara study^13^, posterior uveitis (26.6%) was more prevalent than panuveitis (20.5%) and in the Israeli study^17^, intermediate uveitis (15.2%) was more frequent than posterior uveitis (14.5%). In European studies, anatomical forms in order of frequency were anterior (51.3%), posterior (23.7%), pan- (15.9%), and intermediate uveitis (9.1%); only in the Amsterdam study^23^ was panuveitis (20.4%) more common than posterior uveitis (16.4%) and also in the Swiss study^24^ intermediate uveitis (10.2%) was more common than panuveitis (7.2%), (Table 2). Overall causes of uveitis regardless of anatomical forms are detailed in Table 3.

In Middle Eastern studies, idiopathic uveitis (49.9%) was the most common form of anterior uveitis while Behçet’s disease (13.7%), Fuchs heterochromic iridocyclitis (FHIC) (8.6%), herpes viruses (10.6%), TB (1.9%), and HLA B-27 associated disease (not tested in one of the Saudi Arabian studies^15^) followed in decreasing order. Behçet’s disease was rather frequent in the Ankara report (13%)^13^ and the other study from Turkey (31.3%)^14^ but was uncommon in other Middle Eastern countries. FHIC was reported in several cases in Iran (17.2%)^12^, while herpes and TB were more often encountered in the two Saudi Arabian studies.^15^,^16^ In European studies, idiopathic anterior uveitis was also the most common followed by herpes virus (11.8%), FHIC (10.8%), TB (3.2%), and Behçet’s disease (0.3%). Herpes viruses, TB, and Behçet’s disease (18.9%, 6.3%, and 0.8% respectively) were more common in the Italian study^18^ as compared to other parts of Europe while HLA B-27 associated disorders were more common in Switzerland (15.9%)^24^ and Amsterdam (12.1%)^23^ and FHIC was more frequent in Rome (17%)^21^, Amsterdam (11.3%)^23^, and Switzerland (8.7%)^24^ (Table 4).

In Middle Eastern studies, the most frequently described etiology of posterior uveitis included toxoplasmosis (33.6%), Behçet’s disease (17.4%), idiopathic (16%), Eales disease (4.4%), toxocariasis (3.9%), acute retinal necrosis (3.6%), TB (2.8%), sympathetic ophthalmia (1.9%), and VKH syndrome (1.7%). Highly prevalent conditions were Behçet’s disease in the Ankara (26.3%)^13^ and in the other Turkish (41.2%) study^14^, Eales disease in the Iranian (11.9%)^12^ and Ankara (5%)^13^ studies, and TB in one of the Saudi Arabian studies (29.6%)^16^. Of seven cases of sympathetic ophthalmia, six were reported from Israel^17^ and from a total of 14 cases of toxocariasis, an outstanding number of 11 cases was reported from Iran^12^. VKH syndrome was reported only in the Turkish study (6.2%)^14^. In European studies, toxoplasmosis (49.2%) was similarly the most frequent etiology followed by idiopathic (19.0%), acute retinal necrosis (ARN, 3.0%), Behçet’s disease (2.4%), TB (1.8%), Eales disease (1.6%), toxocariasis (1.4%), and VKH syndrome (0.7%), in decreasing order of frequency. ARN was rather frequent in the Amsterdam (5.6%)^23^, Rome (3.2%)^21^, and Brussels (4%)^22^ studies and so was TB in the Italian (4.1%)^18^ study. The frequency of toxocariasis was prominent in the Rome study (3.2%)^21^ and the same was noted for Behçet’s disease in Brussels (5%)^22^. Eales disease was only reported in the Amsterdam study (9.9%)^23^ (Table 5).

In Middle Eastern studies, Behçet’s disease (44.8%) was the most common cause of panuveitis followed by idiopathic (22.2%), VKH (10.9%), multifocal choroiditis panuveitis (MCP) (3.8%), toxoplasmosis and sympathetic ophthalmia (3.6%), and sarcoidosis (3.4%). Behçet’s disease was frequent in Turkey (53.8%)^14^, Ankara (59.7%)^13^, and Israel (59.2%)^17^, while VKH was common in the Saudi Arabian (31.6 and 12.2%)^15^,^16^ and Iranian (15.2%)^12^ studies. MCP constituted 10.1% of cases in Iran^12^ while sympathetic ophthalmia was reported in 5.1% of cases in Iran^12^ and 9.2% of cases in Israel^17^. In European studies, idiopathic uveitis was the most common form (31.0%) followed by Behçet’s disease (17.7%), toxoplasmosis (11.1%), sarcoidosis (8.7%), VKH (2.6%), and sympathetic ophthalmia (0.3%). Remarkable percentages were reported for Behçet’s disease in Rome (35.3%)^21^, sarcoidosis in Amsterdam (18.8%)^23^ and Brussels (12.5%)^22^, and VKH in Italy (10.6%)^18^ (Table 6).

In Middle Eastern studies, idiopathic intermediate uveitis (82.7%) was most common followed by sarcoidosis (4%), multiple sclerosis (2%), TB (1.6%), and Behçet’s disease (1.2%). Noteworthy percentages were reported for sarcoidosis in Iran (7.3%)^12^ and Turkey (5%)^14^, and for Behçet’s disease in Ankara (11.1%)^13^. In European studies, idiopathic intermediate uveitis was also the most prevalent form (91.3%), followed by sarcoidosis (3.4%), multiple sclerosis (1.7%), TB (0.3%), and Behçet’s disease (0.3%). Sarcoidosis was diagnosed in 9.2% and 2.3% of subjects in the Amsterdam^23^ and Rome^21^ studies, respectively; percentages for multiple sclerosis in the same studies were 5.3% and 1.1% (Table 7).

Regardless of anatomical classifications and considering all causes of uveitis as a whole, in Middle Eastern studies, idiopathic uveitis was found to be most common (41.6%) followed by Behçet’s disease (17.6%), toxoplasmosis (6.3%), herpes viruses (5.5%), FHIC (4.3%), VKH (2.9%), TB (2.8%), HLA B-27 associated disorders (1.9%), sympathetic ophthalmia (1.2%) and sarcoidosis (1.1%). Herpes viruses (11.9% and 16%) and TB (7.6% and 10.5%) were rather frequent in both Saudi Arabian studies^15^,^16^; FHIC was scarce in the Israeli study^17^ but sympathetic ophthalmia was found in a remarkable proportion (3.8%). Other figures worth mentioning were Behcet’s disease in Turkey (32.2%)^14^ and Ankara (26%)^13^, toxoplasmosis (10.1%) and FHIC (6.6%) in Iran^12^, and VKH (7.6%) in one of the Saudi Arabian studies^15^. In European studies, idiopathic uveitis was also most common type overall, followed by toxoplasmosis (9.1%), herpes viruses (6.0%), HLA B-27 associated disorders (5.9%), FHIC (4.9%), Behçet’s disease (2.9%), TB (1.9%), sarcoidosis (1.4%), VKH (1.2%), and sympathetic ophthalmia (0.3%). Idiopathic forms held a high share in the Finnish study (80.8%)^20^. Remarkable prevalence rates were noted for VKH in Rome (2.2%)^21^ and Italy (1.4%)^18^, for toxoplasmosis in Brussels (24.4%)^22^ and Italy (17.7%)^18^, for herpes in Switzerland (12.5%)^24^ and for TB in Italy (7%)^18^. Although Behçet’s disease was not reported at all in the Finnish^20^ study, it was reported in 5.8%, 3.1%, and 5.5% of cases in Rome^21^, Italy^18^, and Brussels^22^ respectively. Sympathetic ophthalmia was reported in 1% (14 cases) in the Rome study^21^. For FHIC, prevalence rates were 8.3% and 6.1% in the Rome^21^ and Amsterdam^23^ studies respectively and HLA-B27 associated disease was found in 12.1% of cases in the latter study and in 15.9% of cases in the Swiss study^24^ (Table 3).

In both Middle Eastern and European studies, toxoplasmosis, herpes viruses, and TB were major infectious agents; they were found in 6.3%, 5.5%, and 2.8% of Middle Eastern and in 9.1%, 6.0%, and 1.9% of European cases, respectively. Remarkably frequent non-infectious causes were Behçet’s disease, FHIC, sarcoidosis, and VKH with prevalence rates of 17.6%, 4.3%, 1.1%, and 2.9% in the Middle East, and 2.9%, 4.9%, 1.4%, and 1.2% in Europe, respectively.

## DISCUSSION

Limitations of epidemiological studies on uveitis stem from a variety of factors including heterogeneity of diagnostic criteria and work-up, lack of uniform classification systems or precise definitions, and the effects of referral and selection bias. These factors render comparison of epidemiological studies from different regions and populations difficult.

Most uveitis studies convey data obtained from tertiary referral centers rather than population-based information. More complicated and severe forms of pan- and posterior uveitis may be over-represented at referral centers; in contrast, primary care and population-based studies tend to report less severe cases such as anterior uveitis.

Average age and male to female ratio did not differ significantly between Middle Eastern and European studies (35.2 versus 39.1 years, and 52% versus 49.4%, respectively).

The most frequent anatomic forms, in order of frequency, were anterior, pan-, posterior and intermediate uveitis in Middle Eastern studies (except for the Ankara study^13^ which was similar to European studies, and the Israeli^17^ study). The most common anatomic forms in European studies included anterior, posterior, pan-, and intermediate uveitis (except for the Amsterdam study^23^ which was similar to Middle Eastern studies). In comparison to the Middle East, posterior uveitis was more common than panuveitis in Europe.

Considering anterior uveitis, idiopathic cases were comparable in Middle Eastern and European studies and were the most prevalent category in all studies. Behçet’s disease in Turkey^14^, FHIC in Iran^12^, and herpes viruses in Saudi Arabia^15^,^16^ and Israel^17^ stood next. In one Saudi Arabian study^16^, TB was rather frequent. As mentioned above, in all European studies, idiopathic cases were the most common group; in the Italian study^18^, herpes viruses, TB and FHIC ranked next, and in the Rome^21^ and Amsterdam^23^ studies, FHIC was frequently reported. In comparison, Behçet’s disease was more frequent in the Middle East while TB, FHIC and herpes viruses were more frequent in Europe.

Regarding posterior uveitis, in the Middle East, toxoplasmosis was the most frequently reported cause in Iran^12^, Ankara^13^ and Saudi Arabia^15^,^16^, while Behçet’s disease in Turkey^14^, and idiopathic forms in Israel^17^ were the most prevalent causes. Noteworthy entities were toxocariasis, FHIC, and ARN in Iran^12^, VKH syndrome in Turkey^14^ and sympathetic ophthalmia in Israel^17^. In European studies, toxoplasmosis was also the most common cause of posterior uveitis in Italy^18^ and Amsterdam^23^; other causes of considerable importance included ARN in Amsterdam^23^, toxocariasis in Rome^21^, and Behçet’s disease in Brussels^22^. Toxoplasmosis and idiopathic cases were more frequent in Europe, while other causes were more common in the Middle East.

Concerning panuveitis, in Middle Eastern studies, Behçet’s disease was consistently the most frequent cause in Iran^12^, Turkey^14^, Ankara^13^, and Israel^17^. In Israel^17^, toxoplasmosis and sympathetic ophthalmia were rather frequent but idiopathic cases were less common. VKH syndrome in Iran^12^ and Saudi Arabia^15^, MCP in Iran^12^, and sarcoidosis in Saudi Arabia^16^ were also common. In all European studies except for Finland^20^, idiopathic cases were most common, but Behcet’s disease in Italy^18^ and Rome^21^, sarcoidosis in Amsterdam^23^, VKH syndrome in Italy^18^, and toxoplasmosis in Finland^20^ were other prominent causes. In general, Behçet’s disease and VKH were more common in the Middle East, while idiopathic cases, toxoplasmosis, and sarcoidosis were more frequent in Europe.

In terms of intermediate uveitis, idiopathic cases held the largest share in both European and Middle Eastern reports. Eye-catching prevalence rates were noted for sarcoidosis in Iran^12^ and Turkey^13^, multiple sclerosis in Saudi Arabia^16^ and Iran^12^, TB in Saudi Arabia^16^ and Behçet’s disease in Ankara^13^. In Europe, other common causes of intermediate uveitis were sarcoidosis and multiple sclerosis in Amsterdam^23^.

Overall, in both geographic regions, idiopathic uveitis was the most common form of intraocular inflammation; in the Middle East, Behçet’s disease and toxoplasmosis, and in Europe, toxoplasmosis and herpes viruses ranked second and third. Behçet’s disease, VKH and sympathetic ophthalmia were more common in the Middle East while toxoplasmosis and HLA-B27 associated disorders were more frequent in Europe. Considering infectious causes, toxoplasmosis in Europe and TB in the Middle East were relatively more prevalent but herpes viruses did not show any prominent differences in prevalence.

Since uveitic entities follow different patterns in different regions and are influenced by a variety of factors, epidemiologic studies can help improve their diagnosis and treatment. Adoption of a universal classification systems and population-based studies in all countries may provide more reliable data for comparisons among different areas.

## Figures and Tables

**Table 1:** Characteristics of uveitis patients in the Middle East and Europe

Middle East							
	Iran	Turkey (Ankara)	Turkey	Saudi Arabia	Saudi Arabia	Israel	Total
Number	544	300	761	488	200	400	2693
Mean age (SD)	32.3 (15.2)	35.7 (−)	35.4 (15.3)	38 (−)	35 (16)	-	35.2
Age range	-	5 – 78	3 – 83	3 – 99	7 – 91	6 – 75	3 – 99
Sex, M/F (M%)	238/306 (44)	162/138 (54)	388/373 (51)	264/224 (54)	120/80 (60)	216/184 (54)	1388/1305 (52)

Europe								
	Italy	Poland	Finland	Italy (Rome)	Brussels	Amsterdam	Switzerland	Total
Number	655	563	120	1417	201	865	558	4379
Mean age (SD)	44.4	40.4	35.4	30.7	36.5	42	44	39.1
Age range	10 – 90	2 – 87	-	-	-	-	5–92	-
Sex M/F (M%)	341/314 (52.1)	263/300 (46.7)	60/60 (50)	667/750 (47.1)	90/111 (45)	433/432 (50)	308/250 (55.2)	2162/2217 (49.4)

**Table 2:** Anatomical types of uveitis in the Middle East and Europe

Middle East							
	Iran	Turkey (Ankara)	Turkey	Saudi Arabia	Saudi Arabia	Israel	Total
Anterior	209 (38.4)	131 (43.5)	400 (52.5)	293 (60)	119 (59.5)	183 (45.8)	1335 (49.6)
Panuveitis	138 (25.4)	62 (20.5)	214 (28.1)	117 (24)	41 (20.5)	98 (24.5)	670 (24.9)
Posterior	101 (18.6)	80 (26.6)	97 (12.7)	54 (11)	27 (13.5)	58 (14.5)	417 (15.5)
Intermediate	96 (17.6)	27 (9)	51 (6.7)	29 (6)	13 (6.5)	61 (15.2)	277 (10.3)

Europe								
	Italy	Poland	Finland	Italy (Rome)	Brussels	Amsterdam	Switzerland	Total
Anterior	380 (58)	251 (44.6)	105 (87.8)	696 (49.1)	-	471 (54.5)	343 (61.5)	2246 (51.3)
Posterior	171 (26.1)	186 (33)	10 (8.2)	313 (22.1)	100 (49.8)	142 (16.4)	118 (21.1)	1040 (23.7)
Panuveitis	85 (12.9)	85 (15.1)	5 (4)	232 (16.3)	72 (35.8)	176 (20.4)	40 (7.2)	695 (15.9)
Intermediate	19 (2.9)	41 (7.3)	0 (0)	176 (12.4)	29 (14.4)	76 (8.8)	57 (10.2)	398 (9.1)

**Table 3:** General causes of uveitis in the Middle East and Europe

Middle east						
	Iran	Turkey (Ankara)	Turkey	Saudi Arabia	Israel	Total
Idiopathic	226 (41.5)	102 (34)	329 (43.2)	86 (43)	158 (39.5)	1121 (41.6)
Behcet’s Disease	47 (8.6)	78 (26)	245 (32.2)	13 (6.5)	61 (15.3)	474 (17.6)
Toxoplasmosis	55 (10.1)	22 (7.3)	36 (4.7)	13 (6.5)	15 (3.8)	169 (6.3)
Herpes	8 (1.5)	9 (3)	22 (2.9)	32 (16)	20 (5)	149 (5.5)
FHIC	36 (6.6)	8 (2.7)	39 (5.1)	7 (3.5)	6 (1.5)	115 (4.3)
VKH	21 (3.9)	3 (1)	9 (1.2)	5 (2.5)	3 (0.8)	78 (2.9)
TB	8 (1.5)	4 (1.3)	3 (0.4)	21 (10.5)	3 (0.8)	76 (2.8)
HLA-B27	3 (0.6)	5 (1.7)	19 (2.5)	3 (1.5)	12 (3)	42 (1.9)
SO	7 (1.3)	2 (0.7)	1 (0.1)	2 (1)	15 (3.8)	27 (1.2)

Europe								
	Italy	Poland	Finland	Italy (Rome)	Brussels	Amsterdam	Switzerland	Total
Idiopathic	302 (46.1)	169 (30)	97 (80.8)	710 (50.1)	60 (29.9)	289 (33.4)	203 (36.4)	1830 (41.8)
Toxoplasmosis	116 (17.7)	-	4 (3.3)	94 (6.6)	49 (24.4)	83 (9.6)	53 (9.5)	399 (9.1)
Herpes	77 (11.8)	-	2 (1.7)	78 (5.5)	4 (2)	30 (3.5)	70 (12.5)	261 (6.0)
FHIC	14 (2.1)	-	0 (0)	118 (8.3)	1 (0.5)	53 (6.1)	30 (5.4)	216 (4.9)
HLA-B27	-	-	-	51 (3.6)	12 (6)	105 (12.1)	89 (15.9)	257 (5.9)
Behcet’s Disease	20 (3.1)	-	0 (0)	82 (5.8)	11 (5.5)	7 (0.8)	6 (1.1)	126 (2.9)
TB	46 (7)	-	2 (1.7)	25 (1.8)	0 (0)	12 (1.4)	- (−)	85 (1.9)
VKH	9 (1.4)	-	0 (0)	31 (2.2)	1 (0.5)	7 (0.8)	4 (0.7)	52 (1.2)
SO	0 (0)	-	0 (0)	14 (1)	1 (0.5)	0 (0)	- (−)	15 (0.3)

FHIC, Fuchs Heterochromic Iridocyclitis; VKH, Vogt-Koyanagi-Harada syndrome; TB, Tuberculosis; SO, Sympathic Ophthalmia

**Table 4:** Etiology of anterior uveitis in the Middle East and Europe

Middle East							
	Iran	Turkey (Ankara)	Turkey	Saudi Arabia	Saudi Arabia	Israel	Total
Idiopathic	109 (52.2)	51 (38.9)	199 (49.8)	155 (52.9)	58 (48.7)	94 (51.4)	666 (49.9)
Behcet’s Disease	0 (0)	17 (13)	125 (31.3)	-	0 (0)	1 (0.5)	143 (13.7)
FHIC	36 (17.2)	8 (6.1)	39 (9.8)	19 (6.5)	7 (5.9)	6 (3.3)	115 (8.6)
Herpes	8 (3.8)	9 (6.9)	22 (5.5)	58 (19.8)	30 (25.2)	15 (8.2)	142 (10.6)
TB	6 (2.9)	2 (1.5)	0 (0)	-	9 (7.6)	3 (1.6)	20 (1.9)

Europe								
	Italy	Poland	Finland	Italy (Rome)	Brussels	Amsterdam	Switzerland	Total
Idiopathic	220 (57.9)	169 (67.3)	91 (86.7)	-	-	155 (32.9)	-	635 (52.6)
Herpes	72 (18.9)	-	2 (1.9)	78 (11.2)	-	24 (5.1)	59 (17.2)	235 (11.8)
FHIC	14 (3.7)	-	0 (0)	118 (17)	-	53 (11.3)	30 (8.7)	215 (10.8)
TB	24 (6.3)	-	1 (1)	-	-	6 (1.3)	-	31 (3.2)
Behcet’s Disease	3 (0.8)	-	0 (0)	-	-	0 (0)	-	3 (0.3)

FHIC, Fuchs Heterochromic Iridocyclitis; TB, Tuberculosis

**Table 5:** Etiology of posterior uveitis in the Middle East and Europe

Middle East							
	Iran	Turkey (Ankara)	Turkey Saudi Arabia		Saudi Arabia	Israel	Total
Toxoplasmosis	55 (54.5)	22 (27.5)	15 (15.5)	28 (51.9)	12 (44.4)	8 (13.8)	140 (33.6)
Behcet’s Disease	0 (0)	21 (26.3)	40 (41.2)	-	0 (0)	2 (3.4)	63 (17.4)
Idiopathic	3 (3)	14 (17.5)	25 (25.8)	-	0 (0)	16 (27.6)	58 (16)
Toxocariasis	11 (10.9)	1 (1.3)	0 (0)	-	0 (0)	2 (3.4)	14 (3.9)
ARN	9 (8.9)	1 (1.3)	0 (0)	-	0 (0)	3 (5.2)	13 (3.6)
Eales	12 (11.9)	4 (5)	0 (0)	0 (0)	0 (0)	0 (0)	16 (4.4)
TB	1 (1)	0 (0)	1 (1)	-	8 (29.6)	0 (0)	10 (2.8)
SO	0 (0)	0 (0)	0 (0)	-	1 (3.7)	6 (10.3)	7 (1.9)
VKH	0 (0)	0 (0)	6 (6.2)	-	0 (0)	0 (0)	6 (1.7)

Europe								
	Italy	Poland	Finland	Italy (Rome)	Brussels	Amsterdam	Switzerland	Total
Toxoplasmosis	103 (60.2)	-	4 (40)	-	39 (39)	69 (48.6)	51 (43.2)	266 (49.2)
Idiopathic	36 (21.1)	-	5 (50)	-	17 (17)	22 (15.5)	23 (19.5)	103 (19.0)
ARN	0 (0)	-	0 (0)	10 (3.2)	4 (4)	8 (5.6)	4 (3.4)	26 (3.0)
TB	7 (4.1)	-	1 (10)	-	0 (0)	2 (1.4)	0 (0)	10 (1.8)
Eales	0 (0)	-	0 (0)	0 (0)	0 (0)	14 (9.9)	0 (0)	14 (1.6)
Toxocariasis	0 (0)	-	0 (0)	10 (3.2)	1 (1)	1 (0.7)	0 (0)	12 (1.4)
Behcet’s Disease	2 (1.2)	-	0 (0)	-	5 (5)	1 (0.7)	5 (4.2)	13 (2.4)
VKH	0 (0)	-	0 (0)	-	1 (1)	0 (0)	3 (2.5)	4 (0.7)
SO	0 (0)	-	0 (0)	-	0 (0)	0 (0)	0 (0)	0 (0)

ARN, Acute Retinal Necrosis; TB, Tuberculosis; SO, Sympathic Ophthalmia; VKH, Vogt-Koyanagi-Harada syndrome

**Table 6:** Etiology of panuveitis in the Middle East and Europe

Middle east							
	Iran	Turkey (Ankara)	Turkey	Saudi Arabia	Saudi Arabia	Israel	Total
Behcet’s Disease	47 (34.1)	37 (59.7)	115 (53.8)	30 (25.6)	13 (31.7)	58 (59.2)	300 (44.8)
Idiopathic	31 (22.5)	15 (24.2)	60 (28.1)	-	13 (31.7)	4 (4.1)	123 (22.2)
VHK	21 (15.2)	3 (4.8)	4 (1.9)	37 (31.6)	5 (12.2)	3 (3.1)	73 (10.9)
MCP	14 (10.1)	0 (0)	7 (3.3)	-	0 (0)	0 (0)	21 (3.8)
SO	7 (5.1)	2 (3.2)	1 (0.5)	-	1 (2.4)	9 (9.2)	20 (3.6)
Toxoplasmosis	0 (0)	0 (0)	12 (5.9)	-	1 (2.4)	7 (7.1)	20 (3.6)
Sarcoidosis	7 (5.1)	3 (4.8)	2 (0.9)	-	6 (14.6)	1 (1)	19 (3.4)

Europe								
	Italy	Poland	Finland	Italy (Rome)	Brussels	Amsterdam	Switzerland	Total
Behcet’s Disease	15 (17.6)	-	0 (0)	82 (35.3)	5 (6.9)	6 (3.4)	0 (0)	108 (17.7)
Idiopathic	28 (32.9)	-	1 (20)	-	24 (33.3)	48 (27.3)	16 (40)	117 (31.0)
Toxoplasmosis	13 (15.3)	-	3 (60)	-	10 (13.9)	14 (8)	2 (5)	42 (11.1)
Sarcoidosis	3 (3.5)	-	0 (0)	2 (0.9)	9 (12.5)	33 (18.8)	6 (15)	53 (8.7)
VHK	9 (10.6)	-	0 (0)	-	0 (0)	1 (0.6)	0 (0)	10 (2.6)
SO	0 (0)	-	0 (0)	-	1 (1.4)	0 (0)	0 (0)	1 (0.3)

VKH, Vogt-Koyanagi-Harada syndrome; MCP, Multifocal Choroiditis Panuveitis; SO, Sympathic Ophthalmia

**Table 7:** Etiology of intermediate uveitis in the Middle East and Europe

Middle East							
	Iran	Turkey (Ankara)	Turkey	Saudi Arabia	Saudi Arabia	Israel	Total
Idiopathic	83 (86.5)	22 (81.5)	47 (92.4)	-	9 (69.2)	44 (72.1)	205 (82.7)
Sarcoidosis	7 (7.3)	0 (0)	3 (5.0)	-	0 (0)	0 (0)	10 (4)
MS	4 (4.2)	0 (0)	0 (0)	-	1 (7.7)	0 (0)	5 (2)
TB	0 (0)	0 (0)	2 (2.5)	-	2 (15.4)	0 (0)	4 (1.6)
Behcet’s Disease	0 (0)	3 (11.1)	0 (0)	-	0 (0)	0 (0)	3 (1.2)

Europe								
	Italy	Poland	Finland	Italy (Rome)	Brussels	Amsterdam	Switzerland	Total
Idiopathic	18 (94.7)	-	-	170 (96.6)	19 (65.5)	64 (84.2)	55 (96.5)	326 (91.3)
Sarcoidosis	0 (0)	-	-	4 (2.3)	1 (3.4)	7 (9.2)	0 (0)	12 (3.4)
MS	0 (0)	-	-	2 (1.1)	0 (0)	4 (5.3)	0 (0)	6 (1.7)
TB	0 (0)	-	-	0 (0)	0 (0)	0 (0)	1 (1.8)	1 (0.3)
Behcet’s Disease	0 (0)	-	-	0 (0)	1 (3.4)	0 (0)	0 (0)	1 (0.3)

MS, Multiple Sclerosis; TB, Tuberculosis
